# Memory Complaint Is a Surrogate for Memory Decline in the Middle-Aged: A Register-Based Study

**DOI:** 10.3390/jcm8111900

**Published:** 2019-11-07

**Authors:** Yah-Yuan Wu, Wen-Chuin Hsu, Yu-Hua Huang, Wei-Min Ho, Yi-Chun Chen

**Affiliations:** 1 Department of Neurology, Chang Gung Memorial Hospital Linkou Medical Center and College of Medicine, Chang Gung University, Taoyuan 333, Taiwan; aa1042@cgmh.org.tw (Y.-Y.W.); wenchuin@adm.cgmh.org.tw (W.-C.H.); yuhua0126@gmail.com (Y.-H.H.); drho@cgmh.org.tw (W.-M.H.); 2 Dementia Center, Chang Gung Memorial Hospital, Taoyuan 333, Taiwan

**Keywords:** subjective cognitive complaints, mild cognitive impairment, young-onset dementia

## Abstract

Memory complaint is one of the earliest symptoms of dementia. The causes and prognosis of memory complaint in the middle-aged population remain largely unknown. We reviewed the register-based data of 2129 patients with memory complaints. Among them, 404 participants were between 40 and 65 years old. The participants were separated into three groups: subjective cognitive decline (SCD), neurodegenerative diseases (ND), and non-neurodegenerative diseases (NND). One-year decline was defined as a decrease of ≥1 on the mini-mental state examination (MMSE). At baseline, 131 participants (32%) were diagnosed with SCD, 141 (35%) with ND, and 132 (33%) with NND. The 1-year cognitive decline rate was higher among patients with ND (36.8%) than in the SCD (7.3%, *p* = 1.3 × 10^−8^) and NND groups (7.6%, *p* = 1.1 × 10^−7^). One-year decline did not differ between the SCD and NND groups. Lower baseline MMSE score predicted increased risk of 1-year cognitive decline (odds ratio (OR) = 1.126, 95% confidence interval (CI) = 1.076–1.178, *p* = 2.52 × 10^−7^). Memory complaint in middle age carried a risk of 1-year cognitive decline, and baseline MMSE is an independent predictor of decline. An initial diagnosis of SCD held the same risk effect for decline as NND. These findings highlighted the necessity for neuropsychological tests in those with memory complaints presenting to the clinic.

## 1. Introduction

Memory complaint is one of the earliest symptoms of Alzheimer’s disease (AD) [1,2,3]. In different community-based studies, the prevalence of memory complaint has ranged from 25% to 50%, depending on sample selection and diagnosis tools [4,5]. The prevalence of memory complaint is higher among individuals with more advanced age, as well as among those with fewer education years [4]. In studies carried out in the memory clinic, the prevalence of memory complaint has ranged from 38% to 46% [6,7]. In community studies, 82% of participants with memory complaint showed no deficits in an objective neuropsychological test [3], while in a hospital-based study, only 46.23% showed no deficits [7], suggesting that seeking medical help is a predictor of cognitive impairment. Memory complaint is also associated with depression and anxiety [8,9]. To improve early diagnosis and provide opportunities for early intervention, researchers must clarify whether memory complaint is associated with dementia risk.

Subjective cognitive decline (SCD) is defined as self-perception of decline in cognitive performance without deficits in objective neuropsychological tests [2,10]. Clinicians often overlook SCD merely as the result of psychiatric disease, such as depression and anxiety [11], or as an age-related phenomenon [4]. However, several studies have reported that patients with SCD have a higher progression rate to dementia or mild cognitive impairment (MCI), with a 1.5- to 3-fold higher risk than healthy controls [3,12,13,14,15,16]. Specifically, in one study, the annual progression rate of SCD to dementia was 2.33%, while that of MCI has been reported to be 5–16% [1,17]. In addition, SCD has been associated with more abnormal findings under magnetic resonance imaging (MRI), including smaller left hippocampus volume [18] and decreased gray matter density over the bilateral medial temporal, frontotemporal, and other neocortical regions [19]. High white matter lesion has also been associated with more cognitive complaints from patients in the SCD group [20,21], and a functional MRI study showed lower functional connectivity in patients with SCD than in healthy controls [22]. A study of cerebrospinal fluid revealed that patients in the SCD group had lower amyloid β and higher tau levels than healthy controls [16,23]. Therefore, SCD should be regarded as a surrogate for neurodegenerative disease [7]. 

However, although elderly patients with both SCD and consistent memory complaint showed a higher incidence of dementia [24,25], only a few SCD studies have focused on patients younger than 65 years old [5,26]. These showed that the SCD prevalence in such patients was 12%, and that SCD was usually associated with psychiatric distress [26]. Young-onset dementia (YOD), defined as dementia with an onset age lower than 65 years, is a significant problem for both patients and society [27]. In one study, the prevalence of YOD was 98.1 per 100,000 individuals between the ages of 45 and 65 years, and it increased with advanced age (33.0 per 100,000 in those aged 45 to 49 years and 166.3 per 100,000 in those aged 60 to 65 years) [28]. In another study, YOD had a broad variety of etiologies, including reversible causes as well as neurodegenerative disease [29]. For instance, traumatic brain injury and substance abuse, like alcoholism, should be causes for concern [30]. Although AD is the most common neurodegenerative disease, accounting for 34% of patients in one study, frontotemporal lobe degeneration (FTLD) accounted for 12% and was more prevalent in patients with YOD than in the elderly [27,28]. In addition, functional impairment was less severe in patients with YOD [30], and the clinical presentation of AD differed between patients with young and old onset. Specifically, psychiatric symptoms in the early stages and faster cognitive decline were noted in patients with young-onset AD [31]. Another investigation involving a 4-year follow-up found that patients with a mean age younger than 65 years have an SCD progression rate to dementia of 2% in that time [7]. It is worth mentioning that the care burden of patients with YOD is higher than that of elderly patients with similar disease severity [32,33].

To date, the predictors of progression and features for clinical diagnosis of SCD in young patients are largely unknown. The present study utilized the registration data of the dementia center of Chang Gung Memory Hospital to review middle-aged patients who sought help for memory complaint.

## 2. Materials and Methods

### 2.1. Study Design and Selection of Participants

**Figure 1:**
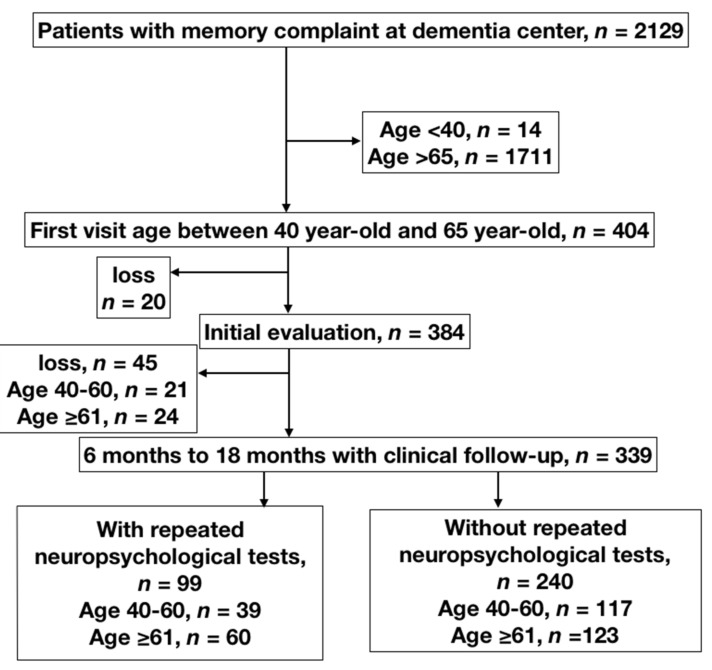
Flowchart of enrolment of patients with memory complaint. Patients with memory complaint (*n* = 404) were enrolled during the 2012–2015 period; 20 were lost to follow-up without any evaluation, 45 completed evaluation but failed to visit 6 months later. Among 404 patients, 339 patients had clinical follow-up over 1 year later. In the 99 patients with observed objective clinical decline, neuropsychological tests were performed again after 6 to 18 months.

This register-based, longitudinal study utilized the dataset of the Dementia Center of Chang Gung Memory Hospital gathered between 2012 and 2015. We selected the patients whose chief complaint was memory impairment (*n* = 2129) and excluded those aged over 65 years or under 40 years [34]. This yielded a cohort of 404 patients (Figure 1). Among these, 339 patients had clinical follow-up over 1 year. In the 99 patients with observed objective clinical decline, neuropsychological tests were performed again after 6 to 18 months. The Institutional Review Board/Ethics Committee (IRB/EC) protocol was approved by the medical ethics committee of Chang Gung Memory Hospital and the ethical approval code was IRB 201900317B0. Given the register-based study, no informed consent was provided in this study.

Progressive functional impairment was clarified through detailed history taking and confirmed using neuropsychological tests. Experienced neurologists or psychiatrists made a diagnosis based on individual clinical criteria. Dementia and its classification were diagnosed by the consensus of two neurologists. 

### 2.2. Diagnosis Criteria

SCD was diagnosed according to the Subjective Cognitive Decline Initiative’s symptomatic definition of pre-MCI SCD [2], which has two main components: (1) compared to their previously normal state, the patient has self-experienced persistent decline in cognitive capacity unrelated to an acute event, and (2) the patient shows normal performance after adjustment for age, gender, and education. In addition, patients were excluded if they had dementia or memory decline caused by medication, medical disease, or psychiatric disease such as depression or anxiety disorder. MCI was diagnosed using Petersen’s criteria [35]. Mood disorders comprised major depression disorder and general anxiety disorder, diagnosed according to The Diagnostic and Statistical Manual of Mental Disorders, 4th edition (DSM-IV) criteria. Dementia was diagnosed according to the criteria for dementia in the DSM-IV-TR. Probable AD was diagnosed according to the National Institute of Neurological and Communicative Disorders and Stroke and the Alzheimer’s Disease and Related Disorders Association (NINCDS-ADRDA) criteria [34,36]. Parkinson’s disease dementia (PDD) was diagnosed according to the consensus criteria of the 2007 version of Clinical Diagnosis of Parkinson’s Disease Dementia, which includes diagnosis of idiopathic Parkinson’s disease and insidious dementia syndrome [37]. Vascular dementia (VaD) was diagnosed using the International Workshop of the National Institute of Neurological Disorders and Stroke (NINDS) and the Association Internationale pour la Recherche et l’Enseignement en Neurosciences (AIREN) criteria, in which the onset of dementia must occur within 3 months of a cerebrovascular event and patients must show rapid or stepwise cognitive deterioration [38]. FTLD was diagnosed using the criteria reported by Neary et al. [39]. Patients presented with symptoms of gradual-onset dysfunction, with inappropriate behavior, personality change, or difficulty with linguistic expression, naming, or word meaning.

### 2.3. One-Year Cognitive Decline

The 1-year cognitive decline was defined when a participant had both objective clinical decline and a reduction of at least one point in the mini mental-state examination (MMSE) within 1 year [40,41]. The middle-aged patients with memory complaint were grouped on the basis of the following baseline characteristics: SCD, neurodegenerative diseases, and non-neurodegenerative diseases. Neurodegenerative diseases were defined as MCI, AD, PDD, and FTLD, while non-neurodegenerative diseases included vascular dementia, dementia caused by structural lesions, and mood disorder (major depression disorder and general anxiety disorder).

### 2.4. Statistical Analyses

Comparisons between groups were conducted using the chi-square test, Fisher’s exact test, and the Student’s t-test where appropriate. ANOVA and post hoc analyses were performed where appropriate. All tests were 2-sided. Logistic regression was performed to identify predictors of 1-year cognitive decline, adjusted for age, sex, education, hypertension, and diabetes mellitus (DM). Statistical significance was defined as a *p* < 0.05. All analyses were performed using IBM SPSS, version 23.0 (IBM Corp, Armonk, NY, USA).

## 3. Results

Among the 404 patients, with an average age of 58.8 ± 6.0 years at the time of first visit, 32% had SCD, 20% had AD, 11% had MCI, 1% had PDD, and 3% had FTLD (Table 1). Patients with non-neurodegenerative disease included those with vascular dementia (6%), mood disorder (21%), and structural lesions (6%) such as alcoholic encephalopathy, normal pressure hydrocephalus, dementia due to herpes simplex virus-related encephalitis, hepatic encephalopathy, and traumatic brain injury.

The patients were diagnosed and classified into three groups: SCD (*n* = 131), neurodegenerative diseases (*n* = 141), and non-neurodegenerative diseases (*n* = 132) (Table 2). In all three groups, there were fewer men than women. Age, hypertension, DM, and education differed significantly between groups. Patients with neurodegenerative diseases were older than patients with SCD or non-neurodegenerative disease. Education was higher in patients with SCD than in patients with neurodegenerative diseases or non-neurodegenerative diseases. Meanwhile, patients with SCD had less hypertension than patients with non-neurodegenerative diseases. Patients with SCD had a lower prevalence of DM than patients with neurodegenerative diseases. Patients with neurodegenerative diseases had a higher 1-year cognitive decline rate than patients with SCD or non-neurodegenerative diseases.

Patients with SCD had the highest total MMSE score, as well as the highest scores in the following MMSE subtypes: orientation, attention calculation, recall, 6-item learning, and 6-item recall, while patients with neurodegenerative diseases had the lowest score (Table 2). Patients with SCD had significantly higher scores than those with neurodegenerative disease in terms of total MMSE (27.9 ± 2.9), orientation, (9.7 ± 0.9), registration (3.0 ± 0.2), attention calculation (4.4 ± 1.0), recall (2.2 ± 0.9), 6-item learning (28.4 ± 2.1), 6-item recall, (5.8 ± 0.5), global clinical dementia rating (5.8 ± 0.5), informant questionnaire on cognitive decline in the elderly (3.8 ± 0.5), and cognitive abilities screening instrument (96.1 ± 8.1) (Table 2). In brief, patients with SCD had significantly higher scores than those with non-neurodegenerative diseases in all tests except registration (Table 2). In 1-year follow-up, the average MMSE score had decreased by 2 points in SCD patients with cognitive decline, while increasing 2 points in SCD without decline (*p* = 3 × 10^−4^). In neurodegenerative diseases, the post hoc analyses comparing AD and MCI showed significant differences in MMSE (*p* = 0.001), orientation (*p* = 2 × 10^−5^), language (*p* = 0.001), global CDR (*p* = 1.4 × 10^−5^), sum of box (p = 9.3 × 10^−5^), IQ CODE (*p* = 0.004), and education year (*p* = 0.01). We did not compare PDD and FTD due to the limitation of small patient number. For non-neurodegenerative disease, mood disorder had a significantly higher score than structure lesion and VaD in MMSE (*p* = 1 × 10^−10^), orientation (*p* = 5 × 10^−16^), registration (*p* = 2 × 10^−7^), attention (*p* = 3 × 10^−5^), recall (*p* = 0.01), language (*p* = 1 × 10^−9^), global CDR (*p* = 2 × 10^−4^) and sum of box (*p* = 5 × 10^−15^).

In the 339 patients with clinical follow-up, 1-year cognitive decline was predicted using the baseline neuropsychological test scores after adjustment for age, sex, education, hypertension, and DM (Table 3). Specifically, the lower baseline MMSE score predicted increased risk of 1-year cognitive decline (odds ratio (OR) = 1.126, 95% confidence interval (CI) = 1.076–1.178, *p* = 2.52 × 10^−7^). The top three strongest predictors were baseline MMSE, orientation (OR = 1.366, 95% CI = 1.225–1.522, *p* = 1.82 × 10^−8^), and recall (OR = 2.208, 95% CI = 1.658–2.941, *p* = 5.93× 10^−8^). The patients’ baseline age, sex, education, hypertension, and DM were not associated with 1-year cognitive decline.

## 4. Discussion

The present study demonstrated a 1-year decline of 7.3% in young patients with SCD, compared with 36.8% in young patients with neurodegenerative disease and 7.6% in those with non-neurodegenerative disease. To our knowledge, this was the first study to focus on longitudinal change in middle-aged patients with memory complaint and to illustrate both etiology and 1-year cognitive decline rate. The 1-year decline rate was significantly higher in patients with neurodegenerative diseases than in the SCD and non-neurodegenerative groups. There was no significant difference in 1-year decline between the SCD and non-neurodegenerative groups. One-year cognitive decline was predicted using the baseline score of neuropsychological tests after adjustment for confounding factors. In this regard, we emphasize the importance of neuropsychological tests for prognosis and prediction in middle-aged patients with memory complaint. 

The present results indicate that AD is the most common causes of YOD [42,43,44] (Table 4), while prior reports have demonstrated that AD, VaD, and FTLD are the most common YOD subtypes [27]. According to a 2003 community study by Harvey et al. [28], the highest proportion of patients with YOD in London comprised those with AD (43%), followed by those with VaD (18%) and FTLD (12%). By contrast, data from a community-based study in Japan in 2006 revealed that VaD (42.5%), followed by AD (25.6%), was the most frequent cause of YOD [45], whereas a memory clinic-based study carried out in Japan between 1997 and 2005 suggested that AD was the most frequent cause of YOD (38.5%), followed by FTLD (21.4%) and VaD (12.6%) [46]. The incidence of VaD has varied across studies and may be strongly impacted by vascular risk factors and socioeconomic status across different regions [47]. The present study suggested that VaD is the second leading cause of YOD, which supports the previous finding [42]. The demographic distribution of middle-aged patients with memory complaint helps clinicians identify abnormal proportions and possible differential diagnosis.

The present register-based study also showed 1-year cognitive decline rate of 37.5% in patients with AD and of 54.5% in those with FTLD, corroborating the previous finding that annual decline rate is greater and baseline MMSE lower in young-onset AD than in FTLD [54]. In the SCD group of the present study, the 1-year cognitive decline rate was as high as 7.3%, suggesting that patients with memory complaint undergo clinical deterioration, even in middle age. The present study classified VaD as a non-neurodegenerative disease, and patients with VaD showed no cognitive decline in the follow-up visit, corroborating a prior finding [41]. In contrast, the majority of decline due to structural lesion occurred in patients with traumatic brain injury.

Aging and lower MMSE have been reported to be predictors for progression from SCD to dementia [55]. However, in young patients, we showed that low baseline MMSE, but not age, was a predictor of 1-year decline. Long-term verbal memory was shown to predict progression from SCD to AD in a hospital-based study [56]. Although the MMSE may suffer a ceiling effect in patients with YOD [57], it is still the most prevalent test in regular practice. The present study provided evidence that the MMSE can be used to predict 1-year decline in middle-aged patients in clinical practice.

To our knowledge, this is the first longitudinal study reviewing the outcomes of middle-age patients with memory complaint. We revealed their baseline characteristics, neuropsychological tests, and heterogenous etiologies. We showed the prognosis and predictors for 1-year cognitive decline and highlight the clinical importance of memory complaint in middle-aged patients. In spite of the above strengths of the study, our study has several limitations. Firstly, as a registration study, not all of the patients had clinical follow-up. Secondly, neuropsychological tests were performed only when objective decline was observed by clinicians, which may lead to underestimation of the 1-year cognitive decline rate. The follow-up rate varied in different disease groups. Finally, this registration did not include comprehensive information, such as family history.

## 5. Conclusions

In conclusion, memory complaint in middle-aged patients had a number of causes in the present study, and it was associated with a risk of further cognitive decline. Baseline MMSE was an independent predictor of 1-year cognitive decline, and SCD carried the same risk of decline as non-neurodegenerative diseases. These findings highlighted the necessity of neuropsychological tests in those with memory complaint who present to the clinic.

## Figures and Tables

**Table 1:** Diagnostic category and frequency of patients with memory complaint.

Diagnosis	Total Enrollment, *n* (%)	1-Year Follow-Up, *n* (%)	1-Year Cognitive Decline, *n* (%)
Subjective cognitive decline	131 (32%)	95 (73%)	7 (7.3%)
Objective cognitive impairment			
Neurodegenerative disease	141 (35%)	125 (89%)	46 (36.8%)
Mild cognitive decline	46 (11%)	40 (87%)	12 (30%)
Alzheimer’s disease	81 (20%)	72 (89%)	27 (37.5%)
Parkinson’s disease dementia	3 (1%)	2 (67%)	1 (50%)
Frontotemporal lobe degeneration	11 (3%)	11 (100%)	6 (54.5%)
Non-neurodegenerative disease	132 (33%)	119 (90%)	9 (7.6%)
Structure lesion ^a^	24 (6%)	23 (96%)	5 (21.7%)
Vascular dementia	24 (6%)	21 (86%)	0 (0%)
Mood disorder ^b^	84 (21%)	75 (89%)	4 (5.3%)
Total	404 (100%)	339 (84%)	62 (18.3%)

^a^ Structure lesion included normal pressure encephalopathy, alcoholic encephalopathy, dementia due to herpes simplex virus-related encephalitis, hepatic encephalopathy, traumatic brain injury, and post-subarachnoid hemorrhage-related encephalopathy; ^b^ Mood disorder includes major depression disorder and general anxiety disorder.

**Table 2:** Clinical, demographic, and cognitive evaluation at baseline.

Characteristic	Total	Subjective Cognitive Decline (SCD)	Neurodegenerative Disease (ND)	Non-Neurodegenerative Disease (NND)	*p* ^SCD–ND^	*p* ^SCD–NND^	*p* ^ND–NND^	*p* ^all^
Number	404	131	141	132				
Age	58.8 ± 6.0	58.4 ± 6.4	60.3 ± 4.7	57.6 ± 6.4	2 × 10^−2^	0.58	1 × 10^−3^	6× 10^−4^
Male, *n* (%)	158 (39.1%)	51 (38.9%)	63 (44.7%)	44 (33.3%)	0.34	0.35	0.06	0.06
Hypertension	115 (28.5%)	30 (22.9%)	40 (28.4%)	45 (34.1%)	0.3	4.4 × 10^−2^	0.31	3× 10^−7^
DM	74 (18.4%)	16 (12.3%)	32 (22.7%)	26 (19.7%)	3.0 × 10^−3^	0.1	0.55	3× 10^−5^
Education (year)	9.7 ± 4.4	10.8 ± 4.2	9.0 ± 4.2	9.3 ± 4.7	2.0 × 10^−3^	1.4 × 10^−2^	0.85	2× 10^−3^
1-year decline	62 (18.3%)	7 (7.3%)	46 (36.8%)	9 (7.6%)	1.3 × 10^−8^	0.617	1.1 × 10^−7^	1× 10^−10^
Neuropsychological test								
Number	384	120	135	129				
MMSE	24.4 ± 6.1	27.9 ± 2.9	21.3 ± 6.3	24.4 ± 6.4	5.1 × 10^−9^	3.0 × 10^−6^	1.8 × 10^−5^	3× 10^−18^
Orientation	8.4 ± 2.4	9.7 ± 0.9	7.2 ± 2.8	8.4 ± 2.4	5.1 × 10^−9^	1.8 × 10^−5^	7.1 × 10^−5^	3× 10^−16^
Registration	2.9 ± 0.5	3.0 ± 0.2	2.8 ± 0.6	2.9 ± 0.5	1.5 × 10^−2^	0.13	0.66	2× 10^−2^
Attention calculation	3.7 ± 1.7	4.4 ± 1.0	3.0 ± 1.8	3.7 ± 1.7	5.1 × 10^−9^	3.4 × 10^−4^	2.3 × 10^−3^	2× 10^−11^
Recall	1.6 ± 1.2	2.2 ± 0.9	0.9 ± 1.0	1.8 ± 1.2	5.1 × 10^−9^	1.3 × 10^−2^	5.1 × 10^−9^	3× 10^−21^
Language	7.9 ± 1.7	8.6 ± 1.0	7.4 ± 2.0	7.8 ± 1.7	2.1 × 10^−8^	6.6 × 10^−4^	0.07	3× 10^−8^
6-item learning	25.0 ± 6.0	28.4 ± 2.1	22.3 ± 7.0	25.0 ± 6.9	7.0 × 10^−9^	1.4 × 10^−3^	9.5 × 10^−3^	5× 10^−9^
6-item recall	4.7 ± 1.8	5.8 ± 0.5	3.7 ± 1.9	4.8 ± 1.9	5.1 × 10^−9^	9.1 × 10^−4^	4.1 × 10^−5^	4× 10^−13^
Global CDR	0.4 ± 0.5	0.2 ± 0.3	0.6 ± 0.4	0.4 ± 0.5	5.1 × 10^−9^	1.7 × 10^−5^	2.6 × 10^−4^	2× 10^−15^
Sum of box	0.5 ± 1.1	3.0 ±3.0	3.0 ± 3.0	2.0 ± 3.4	5.1 × 10^−9^	3.0 × 10^−5^	1.5 × 10^−2^	9× 10^−12^
IQCODE	*n* = 216	*n* = 66	*n* = 89	*n* = 61				
	3.5 ± 0.6	3.2 ± 0.4	3.8 ± 0.5	3.6 ± 0.6	5.1 × 10^−9^	3.0 × 10^−5^	1.5 × 10^−2^	4× 10^−10^
CASI	*n* = 186	*n* = 71	*n* = 58	*n* = 57				
	86.5 ± 12.3	96.1 ± 8.1	78.4 ± 14.5	88.4 ± 9.3	5.3 × 10^−9^	3.8 × 10^−5^	0.14	4× 10^−10^

Comparisons between groups were conducted by the chi-square test, Fisher’s exact test, one-way ANOVA and Student’s t-test where appropriate. Abbreviations: DM = diabetes mellitus; MMSE = mini-mental state exam; CDR = clinical dementia rating scale; IQCODE = informant questionnaire on cognitive decline in the elderly; CASI = cognitive abilities screening instrument; ND = neurodegenerative disease; NND = non-neurodegenerative disease; *p* ^SCD–ND^ = *p* value between patients with SCD and neurodegenerative disease; *p* ^SCD–NND^ = *p* value between patients with SCD non-neurodegenerative disease and; *p* ^ND–NND^ = *p* value between patients with neurodegenerative disease and non-neurodegenerative disease. *p* ^all^ = *p* value of SCD, neurodegenerative disease, and non- neurodegenerative disease.

**Table 3:** Predictors of 1-year cognitive decline in 339 patients.

**Neuropsychological test**	**MMSE**		**Orientation**		**Registration**		**Attention Calculation**		**Recall**	
**Variable**	**OR, 95% CIs**	***p***	**OR, 95% CIs**	***p***	**OR, 95% CIs**	***p***	**OR, 95% CIs**	***p***	**OR, 95% CIs**	***p***
Neuropsychological test	1.126, 1.076–1.178	2.52 × 10^−7^	1.366, 1.225–1.522	1.72 × 10^−8^	1.04, 0.605–1.789	0.885	1.35, 1.144–1.595	4.0 × 10^−4^	2.208, 1.658–2.941	5.93 × 10^−8^
Age	1.012, 0.958–1.069	0.669	1.019, 0.964–1.078	0.498	1.011, 0.959–1.065	0.696	1.014, 0.961–1.070	0.611	1.009, 0.953–1.067	0.765
DM	0.920, 0.410–2.067	0.84	1.009, 0.443–2.301	0.982	0.737, 0.348–1.563	0.427	0.883, 0.406–1.920	0.754	0.669, 0.304–1.474	0.319
Hypertension	1.322, 0.642–2.723	0.449	1.278, 0.617–2.649	0.509	1.199, 0.606–2.370	0.602	1.203, 0.597–2.423	0.605	1.310, 0.642–2.670	0.458
Education	1.037, 0.960–1.120	0.358	1.017, 0.942–1.098	0.658	0.971, 0.906–1.042	0.415	1.013, 0.941–1.090	0.736	1.029, 0.950–1.113	0.487
Sex	0.709, 0.382–1.315	0.275	0.735, 0.393–1.375	0.336	0.756, 0.421–1.358	0.349	0.769, 0.423–1.396	0.387	0.649, 0.348–1.211	0.174
**Neuropsychological test**	**Language**		**Global CDR**		**Sum of box**		**IQCODE ^a^**		**CASI ^b^**	
**Variable**	**OR, 95% CIs**	***p***	**OR, 95% CIs**	***p***	**OR, 95% CIs**	***p***	**OR, 95% CIs**	***p***	**OR, 95% CIs**	***p***
Neuropsychological test	1.312, 1.126–1.531	5.23 × 10^−4^	3.296, 1.828–5.911	6.30 × 10^−5^	2.775, 1.836–4.194	1.0 × 10^−6^	4.254, 2.060–8.783	9.1 × 10^−5^	0.918, 0.880–0.958	8.6 × 10^−5^
Age	1.009, 0.957–1.063	0.747	1.00, 0.947–1.057	0.989	1.009, 0.955–1.067	0.74	1.068, 0.969–1.179	0.186	0.987, 0.909–1.071	0.751
DM	0.841, 0.386–1.834	0.664	0.796, 0.359–1.762	0.573	0.826, 0.366–1.863	0.644	1.360, 0.428–4.322	0.603	0.686, 0.167–2.813	0.601
Hypertension	1.275, 0.632–2.574	0.497	1.482, 0.717–3.063	0.288	1.494, 0.714–3.126	0.286	1.870, 0.682–5.129	0.224	0.544, 0.148–2.003	0.36
Education	1.016, 0.943–1.094	0.681	1.007, 0.934–1.086	0.847	1.025, 0.949–1.108	0.525	0.969, 0.870–1.079	0.57	1.003, 0.868–1.159	0.971
Sex	0.745, 0.410–1.353	0.333	0.748, 0.405–1.381	0.353	0.663, 0.352–1.247	0.202	0.486, 0.202–1.170	0.107	0.668, 0.213–2.091	0.488

Logistic regression model for predictors of 1-year cognitive decline adjusted confounding model of age, sex, education, hypertension and DM: 339 patients had baseline MMSE, orientation, registration, attention/calculation, recall, language, global CDR, sum of box and clinical follow-up; 63 patients had 1-year cognitive decline in 339. ^a^ 178 patients had baseline IQ CODE. 30 patients had 1-year cognitive decline in 178. ^b^ 144 patients had baseline CASI. 23 patients had 1-year cognitive decline in 144. Abbreviations: OR = odd ratio, CIs = confidence intervals, MMSE = mini-mental state exam; CDR = clinical dementia rating scale; IQCODE = informant questionnaire on cognitive decline in the elderly; CASI = cognitive abilities screening instrument.

**Table 4:** Prevalence of young-onset dementia (YOD) in previous studies.

Country	Year	Patient Number	Findings			Reference
AD prevalent			AD	VaD	FTLD	
UK	2003	185	34%	18%	12%	[28]
Japan	2005	34	38%	24%	15%	[48]
Spain	2010	144	42%	14%	10%	[49]
Norway	2019	390	33%	5%	5%	[44]
Japan	2007	185	39%	13%	21%	[46]
UK	2008	54	35%	11%	27%	[50]
Greece	2009	114	27%	6%	25%	[51]
VaD prevalent			AD	VaD	FTLD	
Brazil	2003	141	21%	37%	5%	[47]
India	2004	76	13%	44%	-	[52]
USA	2006	278	17%	29%	3%	[30]
Japan	2006	671	26%	43%	3%	[45]
FTLD prevalent			AD	VaD	FTLD	
Australia	2007	112	12%	3%	19%	[53]
USA	2006	235	1%	6%	13%	[29]

Although prevalence of YOD varied in different studies, AD showed the highest proportion in YOD. Abbreviations: AD = Alzheimer’s disease, VaD = vascular dementia, FTLD = frontotemporal lobe degeneration.
